# Association of serum TNF-α, IL-8 and free light chain with HLA-DR B alleles expression in pulmonary and extra-pulmonary sarcoidosis

**DOI:** 10.1186/s12950-015-0066-3

**Published:** 2015-03-19

**Authors:** Esmaeil Mortaz, Hale Abdoli Sereshki, Atefeh Abedini, Arda Kiani, Mehdi Mirsaeidi, Dina Soroush, Johan Garssen, Aliakbar Velayati, Frank A Redegeld, Ian M Adcock

**Affiliations:** Department of Immunology, Faculty of Medicine, Shahid Beheshti University of Medical Sciences, Tehran, Iran; Division of Pharmacology, Utrecht Institute for Pharmaceutical Sciences, Faculty of Sciences, Utrecht University, Utrecht, the Netherlands; Clinical Tuberculosis and Epidemiology Research Center, National Research and Institute of Tuberculosis and Lung Diseases (NRITLD), Shahid Beheshti University of Medical Sciences, Tehran, Iran; Airways Disease Section, National Heart and Lung Institute, Imperial College London, London, UK; Chronic Respiratory Diseases Research Center, National Research Institute of Tuberculosis and Lung Diseases, Shahid Beheshti University of Medical Sciences, Tehran, Iran; Division of Pulmonary, Critical Care, Sleep and Allergy, University of Illinois at Chicago, Chicago, Illinois USA

**Keywords:** Sarcoidosis, HLA-DR, TNF-α, IL-8

## Abstract

**Background:**

Sarcoidosis is a systemic disease of unknown etiology characterized histologically by the observation of non-caseating granulomas and several immunological abnormalities. Sarcoidosis is a multi-organ disorder which involves formation of granulomas in many tissues including the lungs (pulmonary) and others such as skin, bone, heart (extra pulmonary). Associations between human leukocyte antigens (HLA), the encoded cell surface receptor (HLA-DR) and sarcoidosis have been reported in several studies. Several HLA-DR alleles have been described as potential risk factors for sarcoidosis in distinct ethnic groups however evidence for a relationship between HLA-DR alleles and pulmonary and extra-pulmonary sarcoidosis (EPS) is still scarce. Although the etiology of the disease remains unclear, infectious and environmental factors have been postulated. Inflammatory cytokines and chemokines may play important roles in the pathogenesis of sarcoidosis and serum free light chain (FLC) numbers have been implicated in several immunologic disorders.

**Purpose of the study:**

The aim of the present study was to investigate HLA associations with serum cytokines and FLC in Iranian patients with pulmonary (n = 86) and EPS (n = 46).

**Results:**

We found that among the 16 HLA DRB alleles only *7 and *12 were different in sarcoidosis patients. The levels of TNF-α and IL-8 in pulmonary sarcoidosis patients were higher than in EPS (P < 0.05) whereas the levels of FLC subunits in EPS were higher than in pulmonary sarcoidosis.

**Conclusion:**

This data may suggests a link between HLA-DRB *12 and sarcoidosis in Iranian population.

## Introduction

Sarcoidosis (MIM 181000) is a systemic granulomatous disease that was originally described over 100 years ago [1]. Although any organ system can be involved, pulmonary involvement occurs in about 90% of cases although skin, eye, cardiac, liver and neurologic manifestations are not uncommon. The disorder has been described in all races and in every continent except Antarctica [2]. While many cases may undergo spontaneous regression, one-third of cases are persistent and require therapy. An infectious agent has been suspected as the cause of this disorder but a non-specific etiology has also been identified [1,3].

The inflammatory response in sarcoidosis is characterized by the accumulation of CD4 T cells at the sites of disease activity [4]. A Th1 type response has been suspected because of the granulomatous response and the observation of increased levels of IL-2 and IFN gamma in the disease [5]. These T cells expressed oligoclonal T cell receptors consistent with a response to an environmental antigen [6]. These findings strongly supported an excessive inflammatory reaction or hypersensitivity to some innocuous agent. The utility of corticosteroids and other anti-inflammatory drugs in the treatment of this disorder also support this concept.

A genetic susceptibility to sarcoidosis has been supported by studies demonstrating familial clustering [7]. First-degree relatives have a higher prevalence of disease and familial clustering has been observed in different populations but most noticeably in African Americans [8]. Polymorphisms in a number of candidate genes have been associated with sarcoidosis [9-14].

The clinical presentation of sarcoidosis depends on epidemiological factors such as age, gender and race; the duration of the disease and the anatomical sites involved [15-18]. Although the lungs are involved in most patients with sarcoidosis, recognition of extra-pulmonary sarcoidosis (EPS) requires awareness of the organs most commonly affected, such as the skin and the eyes, and vigilance for the most dangerous manifestations, such as cardiac and neurologic involvement. EPS may be life-threatening and may also affect the therapeutic approach taken [19].

There is evidence that patients with pulmonary plus EPS have greater severity of symptoms, restrictions in daily activities and quality of life and impairment of health status comparing to the patients with isolated pulmonary sarcoidosis [20]. However, there are limited clinical and laboratory parameters that differentiate patients with pulmonary sarcoidosis alone from those patients with accompanying EPS.

Cytokines have been implicated in the pathogenesis of sarcoidosis for many years. In particular, the role of IL-8 and TNF-α have been frequently studied [21-25]. Granulomas in the skin and lungs have recently been described as a source of nuclear IL-33 expression and its expression was strongly correlated with the presence of systemic disease [26]. The data indicated that the local inflammatory milieu within each sarcoidal granuloma is critical for IL-33 expression and that IL-33 expression represents a novel biomarker for systemic involvement of the disease [26].

Serological and molecular analyses of HLA molecules have also been investigated in sarcoidosis. Unfortunately, because of the limitations of study design and techniques used to identify *HLA* molecules (low resolution molecular typing) it has not been possible to reach a consensus on the importance of *HLA* molecules in sarcoidosis [27]. Currently, there is no information on organ involvement and frequency of EPS involvement in sarcoidosis patients from Middle East.

Immunoglobulin free light chains (FLC) can exert various biological functions: enzymatic activity, binding to intracellular and extracellular proteins and cellular interactions [28]. Different inflammatory disorders and autoimmune diseases are accompanied by elevated FLC levels in different body fluids [29-34] and the increased FLC concentrations correlate with relapses of disease and enhanced activity of the immune system [35-39]. Thus, in current study we aimed to investigate whether patients with pulmonary sarcoidosis differ from those with EPS in routinely assessed clinical and laboratory data including HLA-DRB polymorphisms, FLC and TNF-α and IL-8 expression.

## Materials and methods

### Patients

Sarcoidosis patients (n =86) were recruited from Sarcoidosis clinic center from Massih Daneshvari Hospital between January 2012 and August 2014, Tehran-Iran. All patients met the diagnostic criteria for sarcoidosis established by the American Thoracic Society consensus panel [40]. The inclusion criteria consisted of clinical and pathological data and patients with a differential diagnosis were excluded.

Specific phenotypes of sarcoidosis were defined according to the ACCESS group [41]. A summary of the various extra-pulmonary sites in the clinical cohort is shown in Tables 1 and 2. Exclusion criteria were individuals with fungal disease active tuberculosis or who were taking anti-tuberculosis therapy. Pathology slides were reviewed by a trained pathologist at each clinical center and the medical records, chest radiographs and study tests were reviewed by the principal investigator at each clinical center. An interviewer-administered questionnaire was also undertaken by each participant.

**Table 1:** **Characteristics of the study population**

**Organ**	**% (number)**
Pulmonary involvement	63 (86)
Extrapulmonary involvement	40 (46)
Skin	30 (34)
Endocrine	9,3 (8)
Extrathoracic lymph node	11.6 (11)
Eyes	9 (10)
Liver	10.5 (9)
Spleen	10.5 (9)
Cardiac	7 (6)
Ear, nose, and throat	7 (6)
Muscles	14 (12)
Bone/joints	9.3 (8)
Kidney	4.6 (4)

**Table 2:** **Demographic of patients in subjected groups**

**Variable**	**Sarcoidosis**		**Healthy control subjects**
	**Pulmonary**	**Extrapulmonary**	
Number of subjects	86	46	95
Age (mean ± SD) years	39.4 ± 8.8	38.96 ± 9.4	37.7 ± 9.3
Sex			
Female	58.8	26.3	55
Male	27.2	20.7	45
Radiologic stage			
I			
II	2	4	N/A
III	66	19	N/A
IV	18	22	N/A
Current treatment			
Steroid (number)	65	32	N/A
Methotrexate (number)	21	14	N/A
CRP (ng/ml)	13.3 ± 14.6	10.1 ± 3	N/A
Hypercalcemia (number)	46	13	N/A
Anemia (number)	21	3	N/A

### Controls

95 controls were recruited as described previously [42] by random digit dialing (RDD) methods from the same geographic region as the clinical cases. Controls were matched to cases on the basis of age (within 5 years), gender and self-reported race and ethnicity. Controls were excluded if they reported a history of sarcoidosis or medical conditions that made the determination of sarcoidosis uncertain (e.g. granulomatous hepatitis or idiopathic uveitis). This study was approved by the Ethics Committee of Massih Daneshvari Hospital, Medical College of Shahid Beheshti University and the Institutional Review Board of National Research Institute Tuberculosis and Lung Diseases (NRITLD) Medical Center and was in compliance with the national legislation and Declaration of Helsinki guidelines.

### DNA preparation

Heparinized blood was collected from each case and control at the time of the interview and was sent by overnight courier to the DNA core laboratory for DNA isolation and purification. High molecular weight DNA was isolated from non-coagulated blood by detergent lysis and organic extraction. Purified DNA samples were diluted to a standard concentration (1 μg/ml) in 10 mMTris, 5 mM EDTA buffer and being frozen at −70°C until analysed. DNA integrity and concentrations were monitored using agarose gel electrophoresis and ethidium bromide staining.

### Determination of HLA-DRB alleles

Determination of HLA-DR B1 subtypes was performed using single specific primer-polymerase chain reaction (SSP-PCR) methods (Texas BioGene, USA).The PCR products were loaded onto a 0.5% agarose gel and the expression of various alleles was visualized by Gel DOC (Bio-RAD, USA). HLA-DR subtypes were calculated according to the manufacturer’s instructions. Only primers that completely matched the target sequences result in amplified products under the controlled PCR conditions and the presence of an amplified DNA fragment is a positive indication of the existence of an allele-specific sequence in the genomic DNA.

### Quantification of serum cytokines

IL-8 concentrations were quantified by ELISA (BD Biosciences Pharmingen, Breda, The Netherlands) according to the manufacturer’s instructions. Serum levels of TNF-α, IL-6 and IL-1β were also measured by ELISA kits (Invitrogen, USA) according to the manufacturer’s instructions.

### Measurement of FLC in serum

Total lambda (λ) and kappa (κ) FLC concentrations were determined in all sera using an ELISA adapted from Abe *et al.* as described previously [43,44]. In brief, plates were coated o/n with goat anti-mouse IgG (M4280; Sigma, Zwijndrecht, The Netherlands) at 4°C, blocked (1 h; RT) before incubation with mouse anti-human κ or λ Ig-FLC MAb’s (obtained from Dr. A. Solomon, Tennessee, USA). After incubation with different dilutions of samples and standards (The Binding Site, Birmingham, UK), plates were incubated with HRP-labeled goat F(ab’)2-anti-human λ and κ light chain antibodies (AHI1804 andAHI1904, respectively; Biosource, Life Technologies Europe, Bleiswijk, The Netherlands). TMB was used as substrate. At least three data points within the linear range of the standard curve were used per sample to estimate the FLC concentration.

### Statistical analysis

All conditions were performed in triplicate, and all experiments were repeated up to five times. Results are presented as mean ± S.E.M. Data were compared using the unpaired 2-tailed, student’s *t*-test using GraphPad Prism (version 2.01). Results were considered statistically significant when p < 0.05.

## Results

### Analysis of the HLADR *B1-16 alleles in the study population

**Figure 1:**
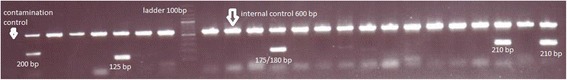
**Representative gel electrophoresis of PCR products.** Detection of allele-specific amplified bands in 0.5% agarose gel by single specific primer-polymerase chain reaction (SSP-PCR).The gel is representative of the gel analysis of all DNA samples. Cont = control subject, EPS = extra-pulmonary sarcoidosis patient and PS = pulmonary sarcoidosis patient. The product size in bp refers to the amplification of a selective allele in that sample. The internal control represents a conserved region of the house keeping gene (provided in kit) and serves as an indication of the integrity of PCR reaction.

To determine whether there were different allelic distributions in sarcoidosis patients and their matched controls, a global test was performed for each allele group of the 16 DRB *1-16 alleles detected. A representative gel of the results is shown in Figure 1. The data was quantified and allelic frequencies are given in Tables 3 and 4. The HLA-DRB1 *7 allele was significantly different between pulmonary (17.7%) and EPS subjects (4.1%, p < 0.05) (Table 3). In addition there was a significant difference in the frequency of the HLA-DRB1 *12 allele between pulmonary (1%) and EPS patients (8.2%, p < 0.05) (Table 3) and this allele was also identified as a risk factor for sarcoidosis. The distributions of the HLA-DRB1 alleles were significantly different between the pulmonary sarcoidosis patients and the EPS patients (P < 0.001, Table 4).

**Table 3:** **Frequency of HLA-DRB1 in control and sarcoidosis patients**

DR B1*	**% patients (n = 86)**	**% controls (n = 95)**	**P-value**
*01	6.1%	10.4%	0.54
*03	18.4%	14.6%	0.55
*04	10.2%	18.4%	0.18
*07	4.1%	17.7%	0.02
*08	--------	5.2%	0.16
*09	--------	1%	1.0
*10	12.2%	5.2%	0.18
*11	36.6%	36.7%	0.87
*12	8.2%	1%	0.04
*13	22.4%	22.9%	0.95
*14	20.2%	12.5%	0.21
*15	32.7%	18.8%	0.06
*16	8.2%	14.6%	0.27

**Table 4:** **Frequency of HLA-DRB1 in pulmonary and extra pulmonary sarcoidosis**

HLA-DRB1*	**Pulmonary**	**Extra-pulmonary**
*01	4	-
*03	4	2
*04	3	-
*07	2	-
*08	-	-
*09	-	-
*10	3	2
*11	19	4
*12	3	-
*13	8	2
*14	8	-
*15	15	4
*16	2	2

### Levels of IL-8, IL-6and IL-1β in serum of pulmonary and extra-pulmonary sarcoidosis

**Figure 2:**
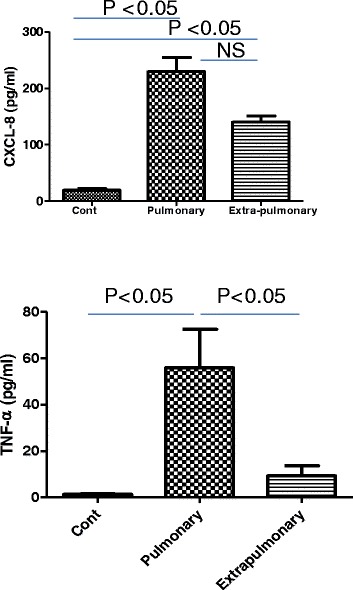
**Serum IL-8 and TNF-α** **levels in sarcoidosis patients.** Serum TNF-αandIL-L8 levels were measured in patients with pulmonary or extra-pulmonary sarcoidosis. Control subjects were age-matched with a negative PPD (purified protein derivative) test and lower angiotensin-converting enzyme (ACE) levels. Data are presented as mean ± SEM (n = 30) in each group except for controls where n = 20). *p ≤ 0.05, **p ≤ 0.01 and ***p ≤ 0.01 compared with control.

The serum levels of IL-8 were significantly increased in both pulmonary and EPS patients compared with control subjects (Figure 2, upper panel). There was no significant difference between the two sarcoidosis groups however. The serum levels of IL-6 and IL-1β were not significantly different between patients with pulmonary sarcoidosis or EPS or between healthy controls (data not shown).

### Levels of TNF-α in pulmonary higher than extrapulmonary sarcoidosis

Serum TNF-α levels in pulmonary sarcoidosis patients were significantly increased compared with control subjects, but no increase was observed in patients with EPS (Figure 2, lower panel).

### Free light chain levels in the serum of pulmonary sarcoidosis and EPS patients

**Figure 3:**
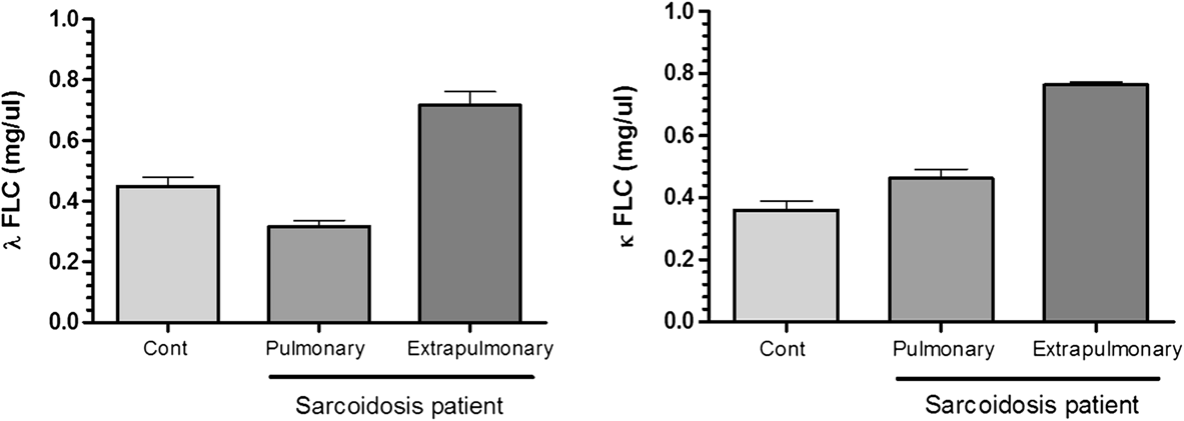
**The levels of free light chain (FLC)** **κand** **λ** **in serum of sarcoidosis patients.** Free light chain concentrations in serum of sarcoidosis patients. Kappa (κ, right panel) and lambda (λ, left panel) FLC concentrations are significantly highly increased in the serum of extra-pulmonary sarcoidosis patients compared with patients with pulmonary sarcoidosis and in control subjects. Results are expressed as means ± SEM of results from 20 control subjects, 30 pulmonary sarcoidosis subjects and 30 extra-pulmonary sarcoidosis patients.

There was a significant increase in serum κ (Figure 3, right panel) and λ (Figure 3, left panel) FLC in patients with EPS compared with control patients. There was no increase in either κ or λ FLC levels in patients with pulmonary sarcoidosis as compared to control subjects. The increase in λ FLC in EPS was also significantly greater than that in pulmonary sarcoidosis patients.

## Discussion

In this study we investigated whether HLA-DRB * sub-typing could distinguish between sarcoidosis patients with pulmonary or extra-pulmonary involvement. We found that among the 16 HLA DRB alleles only *7 and *12 were different between sarcoidosis patients and controls with *12 being identified as a risk factor for sarcoidosis [45]. This study shows also a significant difference between the overall distribution of the HLA-DRB1 alleles between pulmonary sarcoidosis patients and patients with EPS. We further demonstrated increased levels of serum TNF-α and IL-8 in pulmonary sarcoidosis patients, while TNF-α was not increased in EPS subjects. Serum levels of FLCs were higher in EPS patients and not in pulmonary sarcoidosis. This is the first report showing different expression of HLA-DRB sub-types in pulmonary and EPS. The differential presence of pro-inflammatory markers such as TNF-α, IL-8, and FLCs may point to a different pathology in the two sarcoidosis subgroups.

Among the alleles tested, only the expression of the *07 and *12 were significantly altered in sarcoidosis with the frequency of *07 being decreased whereas that of *12 being increased compared with control subjects. It has previously been reported that the presence of the HLA-DR B *7 allele is related to a good disease prognosis [46,47]. Indeed, our patients with this genotype had good response to treatment. In contrast, in Swedish sarcoidosis patients the levels of DHL-DR *7 are increased [48]. The frequency of HLA-DR B*012 expression has been extensively reviewed [49] and the HLA DR-B*12 frequency is similar in our population as in three other populations from UK, Holland and Japan.

Variations in HLA-DRs associations have also been reported in sarcoidosis patients across different ethnic groups. For example, although the frequency of HLA-DRw52 was significantly greater in Japanese sarcoidosis patients compared to control groups there was no significant association with HLA-B antigens [50]. In addition, an association between sarcoidosis and HLA-DR5 has been reported in a German cohort of sarcoidosis patients [51]. This emphasizes that there may be ethnic differences in the expression of these specific alleles in sarcoidosis patients and that there is a need to further analyze other HLA class I and II subtypes in Iranian sarcoidosis patients in future studies.

We also report enhanced levels of serum IL-8 in both pulmonary sarcoidosis and EPS patients. To our knowledge there has only been one previous report showing elevated IL-8 levels in sarcoidosis [52]. Car and colleagues showed that IL-8 levels were significantly increased in bronchoalveolar fluid (BAL) of patients with sarcoidosis [53]. IL-8 is rapidly produced by different cell types upon exposure to inflammatory stimuli such as TNF-α and LPS and is considered as one of the most potent neutrophil chemoattractants in human tissue [54-57]. Recent evidence using blood transcriptomic analysis [58] indicates that neutrophilia is present in sarcoidosis patients as well as in other interferon- and immune-related diseases linked to activation of pattern recognition receptors and to anti-bacterial and -viral defenses. The clinical importance of the increased levels of IL-8 in serum and BAL needs further testing.

In addition, serum levels of TNF-α were increased in patients with pulmonary sarcoidosis but not in those with EPS. TNF-α is a pro-inflammatory cytokine previously shown to play a critical role in the pathogenesis of Th1 responses and to be elevated in sarcoidosis patients [58]. TNF-α plays a significant role in antigen-stimulated, cell-mediated immune responses and in the development of non-caseating granulomas in a variety of diseases [59]. In sarcoidosis, alveolar macrophage-derived TNF-α participates in the induction and maintenance of granulomas [60] and high levels of TNF-α released from alveolar macrophages seem to correlate with disease progression [61]. In addition, BAL levels of TNF-α and IL-6 are significantly higher in pulmonary sarcoidosis patients compared with control subjects [62]. Thus, we were able to recapitulate the BAL findings in relation to TNF-α in blood in our study.

However, the failure to demonstrate increased serum levels of IL-1β and IL-6 in sarcoidosis requires further investigation. This may result from the involvement of distinct cell types present in blood and BAL since these cytokines and chemokines are all stimulated by the same stimuli involving NF-κB activation [63]. This may also explain the differences in TNF-α expression observed between pulmonary sarcoidosis and EPS.

We also report elevated levels of both κ and λ FLC in the serum of EPS patients but not patients with pulmonary sarcoidosis. Sarcoidosis is known as a T cell mediated disease but B-cells may play a role in its pathogenesis particularly in chronic disease [64,65]. It is possible that the release of free light chains is from a distinct set of inflammatory cells in the blood of these patients. A growing body of evidence suggests that serum FLCs could be useful biomarkers in several immunological conditions as they reflect B-cell polyclonal activation including active sarcoidosis [65]. Serum FLC levels are also elevated in lupus [66], rheumatoid arthritis and Sjögren syndrome [37,45,67] where they are also associated with the active state of the disease. Serum FLCs have also recently been reported as biomarkers of systemic sclerosis activity and severity [68,69]. In the current study the expression of κ and λ FLCs was greater in EPS patients compared with patients with pulmonary sarcoidosis. The differential expression of κ and λ FLCs in EPS and pulmonary sarcoidosis requires confirmation in other cohorts and its functional importance needs to be further established. However, we speculate that B cells may play a more significant role in EPS compared to pulmonary sarcoidosis and that these patients may benefit from B cell-directed therapy.

In conclusion, we report for the first time that HLA DRB *12 may be indicative of sarcoidosis and that there is a differential HLA DRB allele usage between patients with EPS and pulmonary sarcoidosis. There were also differences in serum inflammatory markers between these two groups of sarcoidosis patients with patients with pulmonary sarcoidosis having greater levels of TNF-α and IL-8 than EPS and control subjects. Conversely, the expression of κ and λ FLCs was greater in EPS patients compared with pulmonary sarcoidosis patients. Overall, we report a link between HLA-DRB sub-types and inflammation in Iranian patients with sarcoidosis. We suggest that these novel markers should be readily incorporated into current laboratory analysis as they are relatively cheap and rapid, taking less than 48 h, and provide additional parameters for the differentiation of subtypes of sarcoidosis.
